# Effects of hemochromatosis and transferrin gene mutations on peripheral iron dyshomeostasis in mild cognitive impairment and Alzheimer's and Parkinson's diseases

**DOI:** 10.3389/fnagi.2013.00037

**Published:** 2013-08-05

**Authors:** S. Mariani, M. Ventriglia, I. Simonelli, G. Spalletta, S. Bucossi, M. Siotto, F. Assogna, J. M. Melgari, F. Vernieri, R. Squitti

**Affiliations:** ^1^Neurology, University “Campus Biomedico”Rome, Italy; ^2^Department of Neuroscience, AFaR-Fatebenefratelli HospitalRome, Italy; ^3^Medical Statistics and Information Technology, AFaR-Fatebenefratelli Association for the ResearchRome, Italy; ^4^Istituto di Ricovero e Cura a Carattere Scientifico, Fondazione Santa LuciaRome, Italy; ^5^Don Carlo Gnocchi Foundation ONLUSMilan, Italy

**Keywords:** iron, Alzheimer's disease, Parkinson's disease, *HFE*, transferrin

## Abstract

Deregulation of iron metabolism has been observed in patients with neurodegenerative diseases. We have carried out a molecular analysis investigating the interaction between iron specific gene variants [transferrin (*TF*, P589S), hemochromatosis *(HFE)* C282Y and (H63D)], iron biochemical variables [iron, Tf, ceruloplasmin (Cp), Cp:Tf ratio and % of Tf saturation (% Tf-sat)] and apolipoprotein E (*APOE*) gene variants in 139 Alzheimer's disease (AD), 27 Mild Cognitive Impairment (MCI), 78 Parkinson's disease (PD) patients and 139 healthy controls to investigate mechanisms of iron regulation or toxicity. No difference in genetic variant distributions between patients and controls was found in our Italian sample, but the stratification for the *APOE*ε4 allele revealed that among the *APOE*ε4 carriers was higher the frequency of those carriers of at least a mutated *TF* P589S allele. Decreased Tf in both AD and MCI and increased Cp:Tf ratio in AD vs. controls were detected. A multinomial logistic regression model revealed that increased iron and Cp:Tf ratio and being man instead of woman increased the risk of having PD, that increased values of Cp:Tf ratio corresponded to a 4-fold increase of the relative risk of having MCI, while higher Cp levels were protective for PD and MCI. Our study has some limitations: the small size of the samples, one ethnic group considered, the rarity of some alleles which prevent the statistical power of some genetic analysis. Even though they need confirmation in larger cohorts, our data suggest the hypothesis that deregulation of iron metabolism, in addition to other factors, has some effect on the PD disease risk.

## Introduction

It is now fairly well accepted that transition metals, such as iron and copper, are involved in the pathogenesis of some neurodegenerative disorders, like Alzheimer's disease (AD) and Parkinson's disease (PD). Specifically, an excessive iron accumulation has been reported in neuritic plaques, neurofibrillary tangles and in specific brain areas of patients with AD (Castellani et al., 2007). Moreover, abnormal exchange of cortical zinc has been described to link amyloid pathology with neuronal iron accumulation in AD (Duce et al., 2010) and, more recently, some data show that in AD, hippocampus damage occurs in conjunction with ferritin iron accumulation (Raven et al., 2013). This evidence is in disagreement with a recent meta-analysis, reported that iron is not increased in the AD brain (Schrag et al., 2011). However, neuroimaging *in vivo* as well as post-mortem examinations at autopsy have demonstrated that increased nigral iron content in patients with PD is a prominent pathophysiological feature (Dexter et al., 1987; Graham et al., 2000; Kaur and Andersen, 2004; Zecca et al., 2004; Berg et al., 2006; Rhodes and Ritz, 2008; Péran et al., 2010) and different findings suggest that decreased levels of serum ceruloplasmin may specifically exacerbate nigral iron deposition in PD patients (Bharucha et al., 2008; Jin et al., 2011, 2012; Martinez-Hernandez et al., 2011). We recently performed a meta-analysis of the published studies on serum to elucidate the possible role of systemic metabolism of iron in the risk for developing PD. Our results showed no change of iron levels in PD patients, but evidenced a higher heterogeneity that, although reducing the weight of result, stresses the attention on complex homeostasis of iron that play a crucial role in the pathogenesis of PD (Mariani et al., 2013).

The brain iron content increases with advancing age (Zecca et al., 2001) and could lead to enhanced oxidative stress through protein and lipid peroxidation, and DNA oxidation, causing, eventually, cellular and neuronal damage or death (Halliwell, 1992; Salvador et al., 2010). Brain iron homeostasis is regulated by different factors (Levenson and Tassabehji, 2004; Johnstone and Milward, 2010), among which the transferrin (Tf) and the hemochromatosis (*HFE*) proteins seem to play a key role. These proteins, encoded by the *TF* and *HFE* genes (Barry et al., 2005), compete for binding the Tf receptor (Tfr) (Feder et al., 1998). Specifically, *HFE* is a membrane protein which controls iron absorption by regulating the affinity of Tfr on the cell membrane. *HFE* mutations cause hereditary hemochromatosis, a recessive autosomal disorder, with an increased absorption of dietary iron and its consequent overdeposition in tissues and organs (Phatak et al., 2002; Ajioka and Kushner, 2003; Barry et al., 2005). Concerning *TF* gene, there are different genetic variants due to amino acids substitution in the polypeptide chain. The *TF* C is the most common in Caucasian population and it has at least 16 variants (C1–C16). The C1 variant is the most prevalent (95%) and its encoding polymorphic gene has two allelic variants that produce transferrin C2 (proline in position 589 replaced by a serine, P589S). Both *TF* C2 (rs1049296) and the 2 *HFE*, C282Y (rs1800562) and H63D (rs1799945) gene variants, have been investigated as potential risk factors for neurodegenerative disorders. Studies analyzing the association between *HFE* mutations and AD came to different conclusions regarding the hypothesis that H63D mutation may anticipate the disease onset in sporadic AD (Sampietro et al., 2001; Candore et al., 2003). Still other authors evaluated the possible interaction between *TF* C2 and *HFE* C282Y, supporting the hypothesis that iron transport and regulation play a role in AD (Kauwe et al., 2010; Lehmann et al., 2012). Some authors suggested that the combination of *TF* C2 and *HFE* C282Y can lead to an excess of redox-active iron even in mild cognitive impairment (MCI) (Robson et al., 2004). Data available for PD, reported that the common variants in *HFE* might be a risk factor also for this disease (Guerreiro et al., 2006), even though the same authors did not confirm the risk for AD. Taken together these studies, although not always providing concordant results, seem to support the hypothesis that iron metabolism plays a role in some neurological disorders. To improve knowledge about the mechanism of iron regulation and toxicity in living AD, MCI, and PD subjects, a molecular analysis investigating the interaction between iron specific gene variants, iron biochemical assessments and apolipoprotein E (*APOE*) gene risk factor is reported herein.

## Materials and methods

### Subjects

The subject sample consisted of 383 participants: 139 healthy controls (female (F)% 68, age mean ± standard deviation 63 ± 12 years), 139 AD (F% 71, age 73 ± 8), 27 MCI (F% 52, age 71 ± 8) and 78 PD patients (F% 33, age 67 ± 10; Table 1). Both patients and control subjects were recruited by two specialized dementia care Centers in Rome, Italy: the Department of Neuroscience of San Giovanni Calibita—Fatebenefratelli Hospital, and the Department of Neurology of Campus Bio-Medico University. The diagnosis of “probable AD” was posed according to NINCDS–ADRDA criteria (McKhann et al., 1984; Dubois et al., 2007). AD patients had a MMSE score ≤25 (Folstein et al., 1975). PD patients were diagnosed according to the UK PDS Brain Bank Criteria for the diagnosis of PD (Gibb, 1988) and the disease severity was classified on the basis of Unified Parkinson's Disease Rating Scale (UPDRS).

**Table 1:** **Demographic characteristics and mean values ± *SD* (standard deviation), or median value (minimum-maximum) of the biochemical parameters in study in controls, AD, MCI and PD patients**.

	**Controls**	**AD**	**MCI**	**PD**
**Age**	63 (±12)	73 (±8)	71 (±8)	67 (±10)
**sex (%F)**	68%	71%	52%	33%
***APOE* (%ε4)**	11.4%	31.60%	–	–
**Cp (mg/dL)**	27.5 (10–60.2)	26.8 (±5.3) *p* = 0.9	24.4 (±5.1) *p* = 0.1	27.7 (±5.1) *p* = 0.8
**Iron (μg/dL)**	76.3 (11–190)	73.0 (11–441) *p* = 0.9	83.0 (±27.6) *p* = 0.6	74.84 (12–178) *p* = 0.5
**Tf (g/L)**	2.6 (1.6–4.8)	2.4 (±0.4) *p* < 0.001	2.4 (±0.6) *p* = 0.04	2.7 (±0.5) *p* = 0.9
Cp:Tf ratio ^*^ 10^(−2)^	10.3 (5–21.0)	11.4 (±2.7) *p* = 0.002	10.7 (±2.7) *p* = 0.6	10.8 (±2.3) *p* = 0.5
**Tf saturation (%)**	24.1 (1.8–76)	24.4 (2.25–126.0) *p* = 0.469	27.3 (10.9–51.8) *p* = 0.652	21.7 (2.5–58.8) *p* = 0.203

The inclusion and exclusion criteria for MCI were based on previous seminal studies (Albert et al., 1991; Petersen et al., 1999, 2001; Portet et al., 2006), defining elderly persons who do not meet the criteria for a diagnosis of dementia, with objective cognitive deficits, especially in the memory domain. Criteria were as follows: (I) objective memory impairment on neuropsychological evaluation, as defined by performances P1.5 standard deviation below the mean value of age and education- matched controls for a test battery including Busckhe–Fuld and Memory Rey tests; (II) normal activities of daily living as documented by the history and evidence of independent living; and (III) clinical dementia rating score of 0.5. The exclusion criteria for MCI included: (I) mild AD; (II) evidence of concomitant dementia such as frontotemporal form, vascular dementia, reversible dementias (including pseudo-depressive dementia), fluctuations in cognitive performance, and/or features of mixed dementias; (III) evidence of concomitant extra-pyramidal symptoms; (IV) clinical and indirect evidence of depression as revealed by Geriatric Depression Scale scores higher than 13; (V) other psychiatric disorders, epilepsy, drug addiction, alcohol dependence, and use of psychoactive drugs including acetylcholinesterase inhibitors or other drugs enhancing brain cognitive functions; and (VI) current or previous uncontrolled or complicated systemic diseases (including diabetes mellitus) or traumatic brain injuries (Rossini et al., 2008).

The control sample consisted of healthy volunteers with no clinical evidence of neurological or psychiatric disease. Exclusion criteria for both patients and controls were conditions known to affect copper metabolism and biological variables of oxidative stress (e.g., diabetes mellitus, inflammatory diseases, recent history of heart or respiratory failure, chronic liver or renal failure, malignant tumors, and a recent history of alcohol abuse). All individuals included in this study are Caucasian. The study was approved by the local IRB and all the subjects involved or legal guardians signed their informed consent.

### Genotyping

Genomic DNA from fresh whole blood was prepared using the conventional method for DNA isolation (QLAamp DNA Blood Midi kit). Genotyping of SNPs rs1800562, rs1799945, rs1049296 were achieved by the TaqMan allelic discrimination assays from Applied Biosystems Inc. (Foster City CA). The predesigned SNPs genotyping assays ID are C_1085595_10, C_1085600_10, C_7505275_10 (Applied Biosystems). The total reaction volume per well was 20 μL, including 5 ng genomic DNA, 1 μL TaqMan SNP genotyping assay (containing two PCR primers and two dye (VIC or FAM) labeled TaqMan MGB probes) and 10 μL TaqMan Universal PCR Master Mix (Applied Biosystems), according to the manufacturer's manual. PCR was performed at 95°C for 10 min and 40 cycles at 95°C for 15 s and 60°C for 1 min. The samples were amplified, read, and analyzed using the ABI Prism 7900HT Sequence Detection Systemand ABI Prism SDS 2.4 software. Two blank controls in each 96-well-plate were used for the assay quality control. *APOE* genotyping was performed according to establish methods (Hixson and Vernier, 1990).

### Biochemical investigations

Patients' fasting blood samples were collected in the morning and serum was rapidly stored at −80°C. Ceruloplasmin (Cp) was analyzed by immunoturbidimetry assay (Horiba ABX, Montpellier, France) (Wolf, 1982). Iron was analyzed using Ferene, a ligand capable of forming chelates with iron (II), (Higgins, 1981) and transferrin by mixing human serum with the antibody solution and measuring the resulting immune complexes by immunoturbidimetry assay (Skikne et al., 1990). The percentage of transferrin saturation (% transferrin-sat) was calculated by dividing serum iron by the total iron-binding capacity (TBC = Transferrin in mg/dL ^*^1.25) and multiplying by 100. The ferritin was analyzed by immunoturbidimetry assay enhanced with latex (Simo et al., 1994). All reagents were ABX Pentra from Horiba ABX (Montpellier, France). Finally, we measured the Cp:Tf ratio [Cp/Tf ^*^10 (−2)]. All biochemical measures were automated on a Cobas Mira Plus (Horiba ABX, Montpellier, France) and performed in duplicate.

### Statistical analysis

Demographic and clinical characteristics in our patient and control samples were described either in terms of mean ± SD if quantitative, or in terms of proportions. Student's *t*-test and the chi-square (χ^2^) test, or a non parametric test when appropriate, were used to compare separately each diagnosis (AD, MCI and PD) group vs. healthy controls. The effects of both age and sex were considered in all statistical analyses. Diagnosis groups were compared using ANCOVA models adjusting for genetic variable as well as age and sex. A Multinomial Multiple Logistic Regression model was applied, considering the control group as the reference category, to evaluate the effects of the biological and genetic variables on the probability of being AD or PD or MCI. All tests were interpreted at the 0.05 level of significance. SPSS 16.0 software was used for all statistical analyses.

## Results

We screened participants for C282Y (rs1800562), H63D (rs1799945) *HFE* gene and P589S (rs1049296) *TF* gene mutations in order to test their association with AD, MCI and PD. Gene frequencies were in Hardy-Weinberg equilibrium in each group.

The analysis revealed that there was no significant difference in genotype or allele frequencies of C282Y and H63D (Table 2) *HFE* gene variants among AD, MCI and PD subjects vs. healthy controls, though a C282Y mutation higher frequency approached significance in the MCI group (*p* = 0.06; Table 2). The analysis of the *TF* C2 polymorphism revealed that the wild type genotype was carried by 64% of healthy controls vs. 54% of AD patients (*p* = 0.08; Table 2). Finally, the stratification for the *APOE*ε4 allele revealed a significant difference in the frequency of the mutated genotypes of the *TF* gene between *APOE*ε4 carriers and non-carriers (*p* = 0.001; Table 3).

**Table 2:** **Genotype and allelic frequencies for *HFE* (C282Y, H63D) and *TF* (P589S) mutations in controls, AD, MCI, and PD patients**.

		**Controls**	**AD patients**	**MCI patients**	**PD patients**
***HFE* C282Y**					
Wt	GG	137 (98.6%)	138 (100%)	25 (92.6%)	70 (95.9%)
mutation	GA\ AA	2 (1.4%)	–	2 (7.4%)	3 (4.1%)
			*p* = 0.1	*p* = 0.06	*p* = 0.2
allele	G	276 (99.3%)	276 (100%)	52 (96.3%)	143 (98%)
allele	A	2 (0.7%)	–	2 (3.7)	3 (2%)
			*p* = 0.08	*p* = 0.06	*p* = 0.2
***HFE* H63D**					
wt	CC	99 (71.2%)	99 (71.7%)	21 (77.8%)	53 (68.8%)
mutation	CG\ GG	40 (28.8%)	39 (28.3%)	6 (22.2%)	24 (31.2%)
			*p* = 0.4	*p* = 0.5	*p* = 0.5
allele	C	236 (84.9%)	237 (85.9%)	48 (88.9)	130 (84.4%)
allele	G	42 (15.1%)	39 (14.1%)	6 (11.1%)	24 (15.6%)
			*p* = 0.7	*p* = 0.7	*p* = 1
***TF* C2**					
wt	CC	77 (65.3%)	59 (54.1%)	19 (70.4%)	47 (60.3%)
mutation	CT\ TT	41 (34.7%)	50 (45.9%)	8 (29.6%)	31 (39.7%)
			*p* = 0.08	*p* = 0.4	*p* = 0.2
allele	C	194 (82.2%)	163 (74.8%)	45 (83.3%)	121 (77.6%)
allele	T	42 (17.8%)	55 (25.2%)	9 (16.7%)	35 (22.4%)
			*p* < 0.0001	*p* = 0.8	*p* = 0.3

**Table 3:** **Genotype associated with wild type *TF* C2 in AD patients with (*APOE* ε 4+) and without (*APOE*ε 4−) Apoε4 allele**.

***TF* C2**			***p*-value**
	**CC**	**CT\ TT**	
*APOE*ε4+	12	22	
*APOE*ε4−	47	29	0.01

We measured iron, transferrin, ceruloplasmin, and calculated the Cp:Tf ratio and %Tf saturation in the whole study population. Some of these variables were not normally distributed and so we applied a Mann-Whitney non parametric test and these data are presented as median (min-max) in Table 1. We found a significant reduction in the concentration of Tf both in AD and in MCI and a significant increase of the Cp:Tf ratio in AD patients than in controls (Table 1). Conversely, adjusting for age and sex in an ANCOVA model we observed that there was a significative simple effect of the diagnosis factor on Tf values [*p* = 0.008, *F*_(3, 274)_ = 4.055]; in particular only the estimated parameter for AD diagnosis resulted significative (parameter estimate = −0.197, *t* = −2.124, *p* = 0.035), a significant simple effect of age was evident too [*p* = 0.004, *F*_(1, 274)_= 8.210]. The simple effect of diagnosis was significant on Cp:Tf ratio (*p* = 0.014, *F* = 3.603, *df* = 3,255) but it was due to difference between MCI and controls (parameter estimate = −1.666, *t* = −2.217, *p* = 0.028). Also the simple effect of age [*p* = 0.011, *F*_(1, 255)_ = 6.626) and of sex [*p* = 0.002, *F*_(1, 255)_ = 10.060] were significant on Cp:Tf ratio.

Then, we again performed the ANCOVA analysis taking into account factors like diagnosis, age, sex and genetic variants and their possible interaction on concentration of biochemical variables under study. The analysis revealed a significant influence of the interaction between sex and *HFE* on iron concentrations [*p* = 0.038; *F*_(1, 243)_ = 4.3524] and, always on iron concentrations, a significant simple effect of sex [*p* = 0.023; *F*_(1, 243)_ = 5.212], age [*p* = 0.028 *F*_(1, 243)_ = 4.876]. The analysis reconfirmed the previous results on Cp:Tf ratio while the effect of *HFE* was not significant (p > 0.20). The effect of age was significant [*p* = 0.016, *F*_(1, 264)_ = 5.886] on the Tf values and also the effect of diagnosis factor [*p* = 0.008, *F*_(3, 264)_ = 4.038]. Finally, we observed the only significant effect of sex factor on Cp concentrations [*p* < 0.001, *F*_(1, 301)_ = 35.886]. The probability of having AD, PD or MCI was estimated with a multinomial logistic regression model considering the diagnosis as the dependent variable and the biochemical and genetic markers, as well as sex and age, as the independent variables and the control group was set as the reference category. These statistical analyses confirmed age as the main risk factor: the increase of age (1 year) increased of 14% the risk of being AD (*p* < 0.001. OR = 1.14; 95% C.I. 1.07–1.21). Concerning PD, increased of 1 μg/dL of iron (*p* = 0.031; OR = 1.06; 95% C.I. 1.01–1.11), of Cp:Tf ratio (*p* = 0.016; OR = 2.9; 95% C.I. 1.22–6.75) as well as being man instead of woman (*p* < 0.001; OR = 5.20; 95% C.I. 2.14–12.66) resulted in a higher risk of having PD, while the increase of Cp concentration (1 mg/dL) was a protection factor (*p* = 0.042; OR = 0.72; 95% C.I. 0.53–0.99). In MCI, we observed that increasing values of Cp:Tf corresponded to a 4-fold increase of the relative risk of having MCI (*p* = 0.013; OR = 4.27; 95% C.I. 1.374–13.32), and conversely that higher Cp levels resulted as a protection factor (*p* = 0.009; OR = 0.53; 95% C.I. 0.32–0.85).

## Discussion

Several studies investigated the role of the principal variants of *HFE* and *TF* genes on the risk of having neurodegenerative disorders, in particular AD, PD and MCI even though separately. The results reported are often controversial and this inconsistency suggests that, beside the complex genetic etiology, additional interactions can contribute to the disease, as for example, environmental factors, age, sex, and metal ion involvement or oxidative stress. Considering the current literature, we examined the contribution of *HFE* and *TF* mutations in PD, AD and MCI testing if single gene variants have an impact on iron metabolism. Even though our results don't support the hypothesis that these mutations are a genetic risk factor for Italian cohort they have been reported to have a strong effect on the disease risk in several populations (Buchanan et al., 2002; Combarros et al., 2003; Robson et al., 2004). Moreover, a direct significant synergy between the *TF* C2 allele and the GA and AA genotypes of C282Y of the *HFE* gene on the disease risk has been reported by Robson (Robson et al., 2004) and recently confirmed by two additional studies carried out on very large subject samples (Kauwe et al., 2010; Lehmann et al., 2012). Some of ours negative results can be explained on the basis of the fact that the C282Y *HFE* mutation is very rare in the Italian population (Sampietro et al., 2001), and specifically, in our AD sample there was no patient carrier of the homozygous genotype. Interestingly, while this mutation was rare also in the PD sample, the direct comparison between AD and PD revealed that PD patients had a significantly higher frequency. Even concerning these data, literature is controversial, in fact, Guerreiro and colleagues (Guerreiro et al., 2006) described a significant increase of the prevalence of homozygous C282Y carriers in PD patients compared with healthy controls but not in AD, concluding that it could be a risk factor for PD but not for AD in the Portuguese population. This result is confirmed by some (Dekker et al., 2003) but not all studies (Borie et al., 2002; Buchanan et al., 2002).

We found an interaction between *TF* C2 and *APOE*ε4 alleles (Table 3), evidence already described by some authors (Namekata et al., 1997) but not confirmed by others (Marklova et al., 2012) We observed that in AD patients with at least one copy of the *APOE*ε4 allele, the frequency of the CT or TT genotype of *TF* C2 was almost twice as that in the remaining AD non-*APOE*ε4 allele carriers. The underlying mechanisms of this interaction remain unknown but the effect of the combination between *TF* C2 and *APOE*ε4 in AD patients *is a* meaningful question that deserves further studies.

Concerning the biochemical analysis, we observed a significant decrease of Tf concentration in AD and MCI patients compared with healthy control subjects and an increase of the Cp:Tf ratios in AD in line with previous studies (Squitti, 2012). This finding is in line with the derangements of iron metabolism widely described in AD brain and also in MCI (Lavados et al., 2008; Smith et al., 2010). Altered patterns of *TF* gene expression and of Cp levels and activity in the AD brain have been reported (Loeffler et al., 1994, 1996; Castellani et al., 2007). Cp and Tf interact to limit concentrations of labile (“free”) iron and copper species, and thus play an important role in antioxidant defense in serum. More precisely, the Cp:Tf system is involved in counteracting oxidative stress generated in different body districts by regulating the serum capacity to sequester exchangeable transition metals, which are particularly prone to partake in oxidative stress chain reactions (Kozlov et al., 1984; Hubel et al., 1996; Altamura et al., 2009).

The multinomial logistic regression model presented revealed the significant influence of iron and of Cp:Tf values on the probability of having PD, sustaining that an alteration on iron system is involved in the development of this disease. These results demonstrate that iron dysfunction has an effect on the PD risk, even though this diagnosis has not effect on the mean value of iron measured in general circulation, as demonstrated in a recent meta-analysis (Mariani et al., 2013) and confirmed by the ANCOVA results reported in our current study.

The effect of sex on iron levels in relation with PD is complex. In fact, even though in our recent meta-analysis (Mariani et al., 2013) we didn't find a significant effect of being women on iron circulating levels, the replication study reported in the same study (Mariani et al., 2013) pointed out that women had higher Tf serum levels, which can possibly modify iron internalization and tissue storage. In current study, we have again found a sex effect on the disease risk.

The activation of the Cp:Tf antioxidant system appears a mechanism of the early stages of some neurodegenerative diseases. Current result describes a 4-fold increased risk of having MCI in those subjects with higher values of the Cp:Tf ratio. This fact was document in a previous study demonstrating an increment in the ratio of serum copper to non-heme iron levels predicting which subjects with MCI would progress to dementia vs. those that would remain cognitively stable (Mueller et al., 2012).

Higher systemic Cp concentrations seem a protective factor for both MCI and PD subjects and our results about PD are in accord with recent findings reporting a loss of Cp ferroxidase activity in the substantia nigra of PD cases suggesting that intravenous Cp may have therapeutic potential in PD (Ayton et al., 2012). This evidence is consistent with the role of *Cp* that, oxidizing Fe (II) to Fe (III) allows iron binding to Tf and its mobilization from iron tissue storage. More specifically, the result that increased Cp concentrations are a protecting factor for PD is in line with the evidence that CP mutations have been associated with increased deposits of iron in SN (Hochstrasser et al., 2004, 2005).

A result worthy of interest is the effect of interaction between sex and *HFE*. As detailed in the ANCOVA model, the presence on an *HFE* gene mutation (H63D or C282Y) results in an increased absorption of iron in men. A “disease effect” seems essential to explain this relationship, since increased iron levels cannot be ascribed to the *HFE* change alone, but rather it is caused by the synergy between being man and *HFE* change on iron levels, sustaining previous results (Fargion et al., 1992; Niederau et al., 1996; Adams et al., 1997).

Our study has some limitations that need to be taken into account: the small size of the sample, and specifically the small number of MCI subjects included; one ethnic group considered; the rarity of some alleles which prevent the statistical power of some genetic analysis. Even though they need confirmation in larger cohorts, our data suggest that deregulation of iron metabolism, in addition to other factors, has some effect on the PD disease risk.

### Conflict of interest statement

The authors declare that the research was conducted in the absence of any commercial or financial relationships that could be construed as a potential conflict of interest.
